# Advanced imaging findings in stroke-like migraine attacks after radiation therapy (SMART) syndrome

**DOI:** 10.1007/s00259-022-06042-x

**Published:** 2022-11-23

**Authors:** Nico Teske, Nathalie L. Albert, Robert Forbrig, Nina C. Teske, Louisa von Baumgarten, Wolfgang G. Kunz, Joerg-Christian Tonn, Niklas Thon, Philipp Karschnia

**Affiliations:** 1 Department of Neurosurgery, Ludwig-Maximilians-University School of Medicine, Munich, Germany; 2 German Cancer Consortium (DKTK), Partner Site, Munich, Germany; 3 Department of Nuclear Medicine, Ludwig-Maximilians-University School of Medicine, Munich, Germany; 4 Institute of Neuroradiology, University Hospital, Ludwig-Maximilians-University School of Medicine, Munich, Germany; 5 Department of Neurology, Ludwig-Maximilians-University School of Medicine, Munich, Germany; 6 Department of Radiology, University Hospital, LMU Munich, Munich, Germany

## Main text

A female 60-year-old long-term survivor of high-grade glioma presented with progressive migraine-like left-sided cephalgia and expressive aphasia for three days. Ten years ago, a left temporo-occipital *IDH*-wildtype glioblastoma was treated with radiochemotherapy per EORTC 26981/22981 (TMZ/RT → TMZ; 60 Gy in 30 fractions) resulting in complete remission on follow-up imaging (**A**). On admission, repetitive EEG was negative for patterns on the ictal-interictal continuum. Brain MRI revealed faint gyriform contrast-enhancement (arrowheads) and enlarged vessels (arrows) in gadolinium-enhanced T1-weighted imaging, left temporo-occipital edema adjacent to radiation-induced white matter-hyperintensity on T2-weighted imaging with corresponding diffusion restriction, and temporo-occipital elevation of the left cortical blood volume (CBV) of the previously irradiated left temporo-occipital brain region in comparison to the contralateral hemisphere in perfusion MRI sequences (5.6 mL/100 g vs. 3.1 mL/100 g; **B**). [^18^F]fluorethyltyrosine ([^18^F]FET)-PET detected corresponding gyral tracer uptake with moderate signal intensity and increasing time-activity-curves, not typical for tumor recurrence (**C**) [1]. Diagnosis of *stroke-like migraine attacks after radiation therapy* (SMART) syndrome was given [2]. Under supportive therapy, symptoms and imaging abnormalities fully resolved within 6 weeks (**D**). ([^18^F]FET)-PET 1 year after symptoms showed complete regression of the gyral tracer uptake. SMART syndrome represents a rare, delayed complication of brain-directed radiotherapy involving impaired cerebrovascular autoregulation [3]. Symptoms are characteristically transient in nature, and diagnosis rests upon distinct clinico-radiographic findings as well as exclusion of differentials. To our knowledge, this is the first case demonstrating [^18^F]FET-PET imaging in a patient with ongoing SMART syndrome, which could potentially improve diagnostic accuracy by exclusion of important differentials such as tumor recurrence.
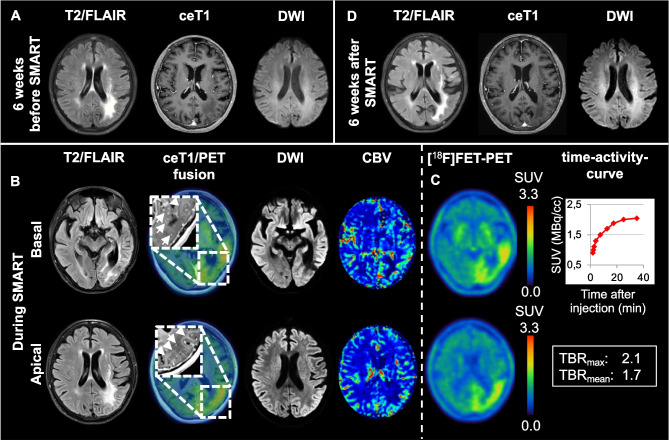


## Data Availability

All data can be made available upon reasonable request to the corresponding author.
